# Comprehensive Assessment of the Risk of Developing Coronary Artery Aneurysm in Kawasaki Disease: A KawaCOR Score Study

**DOI:** 10.3390/life16040607

**Published:** 2026-04-07

**Authors:** Stasa Krasic, Srdjan Pasic, Sanela Nikolic Tepic, Gordana Petrovic, Tijana Djeric, Sergej Prijic, Adrijan Sarajlija, Vladislav Vukomanovic

**Affiliations:** 1 Cardiology Department, Mother and Child Health Institute of Serbia, 11070 Belgrade, Serbia; 2 Faculty of Medicine, University of Belgrade, 11000 Belgrade, Serbia; 3 Immunology Department, Mother and Child Health Institute of Serbia, 11070 Belgrade, Serbia; 4 University Clinical Centre of the Republic of Srpska, 78000 Banja Luka, Bosnia and Herzegovina; 5 Clinical Genetics Outpatient Clinic, Mother and Child Health Institute of Serbia, 11070 Belgrade, Serbia

**Keywords:** kawasaki disease, coronary artery aneurysm, predictive score, KawaCOR score

## Abstract

**Background:** The early identification of high-risk patients is crucial in stratifying treatment algorithms for Kawasaki disease (KD). Our study aimed to develop a new scoring system to predict the risk of developing a coronary artery aneurysm (CAA) and the persistence of giant CAA during follow-up. **Methods:** A retrospective cohort study included 151 patients treated at our institute for KD between 2011 and 2025. **Results:** A total of 25 patients (16.5%) developed CAA, while, in the follow-up period, aneurysms were registered in nine patients. Based on the values obtained from the univariate analysis, a scoring system was developed. It included age <6 months, IVIG treatment for >7 days, refractory KD, leucocytosis (>17 × 10^9^), neutrophilia (>16 × 10^9^), thrombocytosis (>400 × 10^9^), anaemia (<103 g/L), hypoproteinaemia (<54 g/L), and hypoalbuminaemia (<32 g/L). Patients with a score ≥5 had an almost nine-fold higher risk of developing CAA (OR 8.7, 95% CI 3.4–22.6; *p* < 0.001), while, if the score was ≥8, the risk for a chronic giant aneurysm was 71 times higher (OR 71.5, 95%CI 8.5–597.7; *p* < 0.001). Based on the ROC curve, a score ≥5 has 99% sensitivity and 70% specificity for the development of CAA (AUC = 0.87). A score ≥7 has 100% sensitivity and 85% specificity for the development of giant aneurysms (AUC = 0.87). **Conclusions:** The KawaCOR score is the first scoring system in our region specifically designed to predict the development of CAA and acute and chronic giant CAA.

## 1. Introduction

Kawasaki disease (KD) is an acute, self-limited vasculitis affecting medium-sized arteries that mainly affects infants and young children, with an incidence of 4.5 to 9 per 100,000 children under 5 in Europe [1,2]. Coronary artery lesions (CAL) are the most significant complication and the leading cause of acquired paediatric heart disease in developed countries. CAL in KD is associated with higher risks of impairment in coronary thrombus formation, coronary artery stenosis, myocardial infarction, and sudden death even years after the acute illness [1]. Approximately 30% to 50% of patients with KD develop transient coronary artery dilation in the acute stage. Without treatment, almost 25% of patients develop a coronary artery aneurysm (CAA), while therapy with intravenous immunoglobulin (IVIG) reduces their incidence to 3–5% [3]. However, 11–20% of patients are resistant to a first line of IVIG and show a higher risk of CAL [4,5].

Early identification of high-risk patients is crucial for stratifying treatment algorithms for KD and selecting patients at risk for severe disease who would benefit from the intensification of first-line treatments (Supplementary Figure S1). Since 1991, several scores have been developed to identify indications for IVIG treatment (e.g., the Harada risk score) and to predict IVIG resistance (e.g., the Kobayashi, Egami, and Sano risk scores). These scores demonstrate good sensitivity (77–86%) and specificity (67–86%) in the Japanese population, with a notable lack of sensitivity and poor predictive ability in North American, European, and other Asian populations [4]. In North America, baseline CA Z-scores, particularly when combined with age at fever onset, were the most predictive risk factors for the occurrence of CAA within the first 2 to 8 weeks of illness [6,7]. In the European population, Piram et al. presented the Kawanet score, which predicts secondary treatment after initial IVIG and comprises hepatomegaly, ALT ≥ 30 IU/L, lymphocyte count < 2400/mm^3^, and time to treatment < 5 days. The model achieved its best sensitivity (77%) and specificity (60%) with one point per variable and a cutoff of two points or greater, yielding an area under the curve of 0.725. Unfortunately, the absence of CA Z-scores in Kawanet limited the accuracy of the analysis of cardiac complications [4]. Modified Kawanet scores 1 and 2 (a combination of the Kawanet score and echocardiography) had higher sensitivity and specificity than the Kawanet score alone, enabling the early identification of children with KD requiring second-line treatment in multi-ethnic European populations [5]. These scores do not predict the risk of CAA development or persistence during follow-up.

Notably, their limited generalizability across populations and their failure to predict long-term CAA persistence have prompted the development of a new score for Southeastern Europe.

The primary aim of our study was to develop and validate a new scoring system to predict the risk of developing CAA and giant CAA during acute KD. Secondary aims were to identify independent and dependent risk factors for the development of CAA in acute KD and to investigate the persistence of giant CAA during follow-up.

## 2. Methods

### 2.1. Study Design and Study Population

A retrospective cohort study included 151 patients (97 boys, 34, IQR 19–53 months) treated at the Mother and Child Health Institute of Serbia who were diagnosed with KD and treated between 2011 and 2025. The diagnosis was made in accordance with the American Heart Association (AHA) criteria [2]. All included patients had complete laboratory and echocardiographic data at specific time points (e.g., at initial diagnosis, after IVIG treatment, and at follow-up). Patients with incomplete data and with other inflammatory diseases were excluded from the study. The entire cohort was divided and compared with respect to the presence of CAA in both the acute and chronic phases, as well as its size (mild, moderate, or giant). Patients with multisystem inflammatory syndrome in children (MIS-C) that was temporally associated with COVID-19 were excluded from the study.

### 2.2. Data Collection

All data were collected from the electronic medical system and the available medical documentation. The following data were collected: demographic (sex, date of birth, body surface area); clinical (duration of fever before admission and IVIG treatment, presence of specific signs and symptoms, Kobayashi score, and IVIG response); laboratory parameters (complete blood count, inflammatory markers, and biochemical parameters); and echocardiographic parameters (presence of CAA, Z-scores of coronary artery dimensions, left ventricular function, presence of pericardial effusion).

Blood samples were collected before and after IVIG treatment and before discharge.

**Figure 1:**
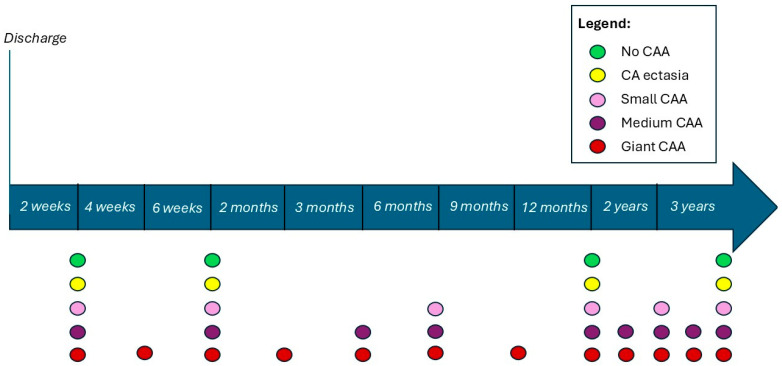
Echocardiographic follow-up in patients with Kawasaki disease after hospital discharge. Abbreviation: CAA—coronary artery aneurysm.

Echocardiography was performed in all patients during the first 10 days of illness (5th to 10th day after fever onset), at discharge, and during the follow-up period. Dimensions of the left main (LM), left anterior descending (LAD), left circumflex (LCx), and proximal right coronary arteries (RCA) were measured using a standardised protocol. Dimensions were adjusted for body surface area and expressed as a Z-score. The classification of CAL was based on AHA criteria [2]. According to our institutional protocol, during follow-up, the frequency of echocardiographic examinations depended on the presence of CAA (Figure 1).

The follow-up period was defined as the interval between the acute KD phase (from hospital discharge) and the most recent echocardiographic examination. In patients who underwent surgery, preoperative echocardiography was considered.

### 2.3. Treatment

Patients received IVIG (2 g/kg over 24 h) and high-dose oral aspirin (30–50 mg/kg per day) until their temperature remained normal for at least 72 h. They then continued low-dose aspirin (3–5 mg/kg per day) for 2 months from the onset of the disease, or longer if CAL was present. Patients with IVIG resistance, defined as persistent or recurrent fever (temperature > 38.0 °C) for at least 36 h, received a second dose of IVIG or methylprednisolone at 30 mg/kg over 2 to 3 h, once daily for 1 to 3 days. A biological agent, specifically the monoclonal antibody infliximab targeting TNF-α, was additionally administered to patients with progressive coronary artery dilation despite prior therapy. Patients who were not treated with IVIG were excluded from the study.

### 2.4. Design of the Kawasaki Disease Coronary Artery Aneurysm (KawaCOR) Score

Based on the final multivariable logistic regression model, a risk score, termed the KawaCOR score, was developed. The primary outcome of the score is the identification of values that predict the development of acute, giant, and chronic CAA.

The entire cohort was divided and compared with respect to the presence of CAA in both the acute and chronic phases, as well as its size (mild, moderate, or giant). After defining the median values of the significantly different numerical variables for each group, the cohort was divided into two groups: those with values below the median and those with values above the median. After splitting patients into groups, binomial and multinomial logistic regression analyses were used to examine the influence on the development of acute, chronic, and giant CAA. If the logistic regression analysis showed a significant impact, the parameters were included in the KawaCOR score. For each independent predictor identified in the final model, one point was assigned for the presence of the risk factor. The total KawaCOR score was the sum of points for all present risk factors.

The ROC curve was used to define cutoff values for the KawaCOR score, which predicted the likelihood of developing acute CAA, chronic CAA, and giant chronic CAA. Validation of the score was performed using the Hosmer–Lemeshow test, sensitivity, and specificity.

### 2.5. Statistics

Basic (descriptive) statistics included mean values, standard deviations, medians, and interquartile ranges (IQRs) for monitored parameters. Furthermore, differences in the distribution of specific features among the tested groups were assessed using the χ^2^ or Fisher’s exact test. The normality of the distribution of numerical variables was tested using the Shapiro–Wilk and Kolmogorov–Smirnov tests. The comparison between the groups was performed using Student’s *t*-test, ANOVA, the Mann–Whitney test, and the Kruskal–Wallis test. Student’s *t*-test and Wilcoxon’s test were used for the dependent variables. We compared patients based on whether they developed CAA, whether they developed chronic giant CAA, and the CAA size. Binomial and multinomial logistic regression analyses were used to model the relationships between the binary dependent variable and the independent variables. Cutoff values for the KawaCOR score were determined using ROC curve analysis. The Hosmer–Lemeshow test was used to assess goodness of fit for logistic regression models in risk prediction.

Given the retrospective design of the study and the inclusion of all eligible patients during the study period, a priori sample size calculation was not feasible. Therefore, a post hoc power analysis was performed using GPower (version 3.1). A two-tailed z-test for the difference between two independent proportions was used to assess the primary outcome (development of coronary artery aneurysm), with an alpha level of 0.05.

All statistical methods were significant if the *p*-value was ≤0.05. Data processing was performed using SPSS Statistics 25.0 for Windows 10.

## 3. Results

### 3.1. Admission Parameters

Between 2011 and 2025, 151 patients were treated at our institute, including 97 boys (64.2%), with a median age of 34 months (IQR 19–53 months; min–max 2–600 months). Kawasaki disease in the first year of life was managed in 25 patients (14 of whom were younger than 6 months), while 22 patients were over 6 years old. The admission date was around day 6 from fever onset (IQR 4–8 days; min–max 1–21). The Kobayashi score on admission was estimated at 2 (IQR 0–3; min–max 0–7). Refractory KD was observed in 38/151 patients (25%).

With a total sample size of 151 patients, the post hoc power analysis demonstrated that statistical power of 96.4% was achieved to detect a significant difference in the development of CAA (Supplementary Figure S3).

### 3.2. Acute Coronary Artery Aneurysm

A total of 25 patients (16.5%) developed CAA. At initial examination, 10 patients had coronary artery ectasia, while 19 had CAA. An additional five patients developed aneurysms during hospitalisation, and one patient developed an aneurysm after disease recurrence. The presence of coronary artery ectasia did not influence the development of CAA (*p* = 0.6).

The incidence of CAA did not differ by gender (17 boys and 8 girls; *p* = 0.7) or by muco-cutaneous manifestations (*p* > 0.05). The estimated Kobayashi score at admission did not influence the development of CAA (*p* = 0.6). During infancy (25 patients), the frequency of CAA was significantly higher (9/25 patients; *p* = 0.004), particularly among those younger than 6 months (8/14 patients; *p* < 0.001), compared with the remainder of the cohort. In patients with CAA, IVIG initiation was later (9, IQR 6–15 days) than in patients without CAA (6, IQR 6–8), *p* = 0.002. Additionally, they had higher white blood cell (WBC) counts, absolute neutrophil counts (ANC), and platelet counts, whereas the concentrations of haemoglobin (Hgb), protein, and albumin were significantly lower than those of the rest of the cohort (Table 1).

By dividing the patients into two groups depending on the median of the obtained values, univariate regression analysis showed a higher risk of developing CAA in the following patients: those younger than 6 months, those with IVIG initiation after 8 days of fever onset, and those with Hgb < 103 g/L, platelet count > 400 × 10^9^, WBC count > 17 × 10^9^ and protein level < 54 g/L. Meanwhile, independent risk factors were age up to 6 months, IVIG initiation after more than 8 days, and protein < 54 g/L (Table 2).

### 3.3. Giant Coronary Artery Aneurysm

A total of 13 patients developed giant aneurysms during hospitalisation, while nine of them (9/13) had refractory KD (*p* = 0.001). Compared with the rest of the cohort, patients who developed giant CAA had significantly higher WBC and ANC counts, while their Hgb, protein, and albumin concentrations were lower (Table 1). The univariate regression analysis showed a higher risk of developing CAA for patients with the following: WBC count > 21 × 10^9^, ANC >16 × 10^9^, Hgb < 103 g/L, protein < 54 g/L, and albumin < 32 g/L. Meanwhile, refractory KD and Hgb < 103 g/L were independent risk factors for the development of giant CAA (Table 3).

At discharge from the hospital, 16 patients had CAA, 13 had giant aneurysms, one had a moderate aneurysm, and two had mild aneurysms. Giant aneurysms were isolated (on one CA) in six patients, while, in others, they were combined.

### 3.4. Chronic Coronary Artery Aneurysm

The follow-up period was 22 months (IQR 6.8–53.4; min–max 6–214 months). At the last echocardiographic examination, aneurysms were registered in nine patients, with five giant, two moderate, and two mild. Of the five patients with residual giant aneurysms, three had IVIG initiation after 12 days of illness, i.e., IVIG initiation was significantly later (*p* = 0.003), and they had less frequent typical KD (1/5 pts, *p* = 0.04) but frequent refractory KD (4/5 pts, *p* = 0.014). These patients had significantly lower albumin levels on admission than the rest of the cohort, as well as significantly higher WBCs and ANCs (Table 1). WBC > 30 × 10^9^/L, ANC > 23 × 10^9^/L, and albumin < 23 g/L increased the risk for chronic giant CAA (Table 4).

**Figure 2:**
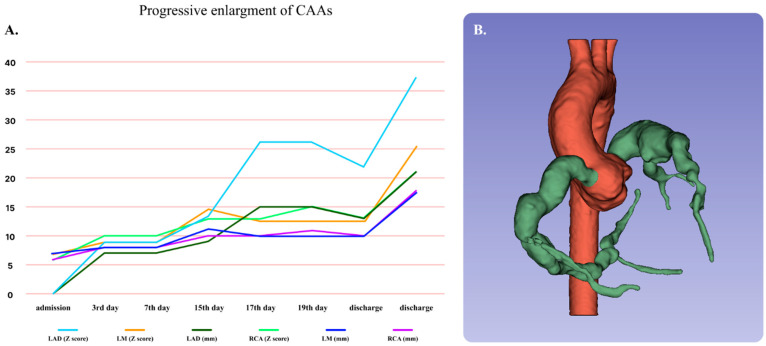
Progressive enlargement of the coronary arteries—LM, LAD, and RCA aneurysms—during acute KD and at 16 months after presentation are shown in the graph as absolute numbers and Z-scores (**A**); 3D reconstruction of the control CT angiography of our patient with progressive giant CAA at 16 months after acute illness (**B**). The first CT angiography was performed 4 months after acute illness. Abbreviations: LM—left main, LAD—left anterior descending, RCA—right coronary artery, KD—Kawasaki disease, CAA—coronary artery aneurysm.

Calibre pseudonormalisation of CA was observed in 16 patients, while, in four patients, the CAA size decreased to mild and moderate (Supplemental Figure S2). In four patients with giant CAA, pseudonormalisation of the diameter was observed. Aneurysm progression occurred in one patient during the 18-month follow-up period (Figure 2A). The patient had an atypical form of Kawasaki disease, with onset at the age of 11 and the development of giant aneurysms on the sixth day of illness. CT coronary angiography 16 months after the acute disease showed giant aneurysms of the LM, LAD, and RCA, without localised stenosis, which were approximately 10 mm larger than on the previous CT scan conducted 12 months before (Figure 2B).

During the follow-up period, CA stenoses were observed in two patients with giant aneurysms—a 7-year-old girl (5 years after KD treatment) and a 7-year-old boy (6.5 years after KD treatment)—who underwent surgical treatment due to ischaemic changes on stress magnetic resonance imaging and cardiac scintigraphy.

### 3.5. KawaCOR Score

Based on the values obtained from the univariate analysis, a scoring system was developed (Table 5).

**Table 5:** KawaCOR score.

Scoring System	KawaCOR Score
**Age**	
**<12 months**	1 point
**<6 months**	2 points
**White Blood Cell Count**	
**>17 × 10^9^**	1 point
**>21 × 10^9^**	2 points
**>32 × 10^9^**	3 points
**Absolute Neutrophil Count**	
**>16 × 10^9^**	1 point
**>23 × 10^9^**	2 points
**Haemoglobin < 103 g/L**	1 point
**Platelets > 400 × 10^9^**	1 point
**Protein < 54 g/L**	1 point
**Albumin < 32 g/L**	1 point
**Duration of Illness > 8 days**	1 point
**Refractory Kawasaki Disease**	1 point

Patients in the CAA group had a significantly higher score (5, IQR 4–7 vs. 2, IQR 0–3; *p* < 0.001) compared to patients without CAA, as well as patients with giant CAA (7, IQR 4–8 vs. 2, IQR 1–4; *p* < 0.001), in the acute phase of the disease, and in the follow-up (8, IQR 5–11 vs. 2, IQR 0–4; *p* = 0.001) compared to patients without CAA and with mild and moderate aneurysms. According to the univariate regression analysis, the cutoff points for predicting acute CAA, giant acute CAA, and chronic giant CAA were ≥5, ≥7, and ≥8, respectively (Table 6). Based on the ROC curve, a score ≥ 5 has 99% sensitivity and 70% specificity for the development of CAA (AUC = 0.87). A score ≥ 7 has 100% sensitivity and 85% specificity for the development of a giant aneurysm (AUC = 0.87).

An increase of one in the score increases the risk almost two-fold for the development of CAA (OR 1.9, 95%CI 1.5–2.5; *p* < 0.001), as well as a giant aneurysm, in the acute phase (OR 1.97, 95%CI 1.4–2.7; *p* < 0.001; Hosmer–Lemeshow test 0.5) and during follow-up period (OR 2.3, 95%CI 1.3–4.06; *p* = 0.002; Hosmer–Lemeshow test 0.75). Our score calculator can be find on the link: https://www.imd.org.rs/kawacor-score/.

## 4. Discussion

In addition to standard risk factors, some scores include other laboratory findings, such as platelet counts, haemoglobin, lymphocyte counts, albumin, erythrocyte sedimentation rates, ALT, AST, and total bilirubin [1,2,3,4,5,6,7,8,9]. These scores predict IVIG resistance and CAA development only during the acute phase and short-term follow-up. Our study introduces a novel scoring system that reliably predicts the risk of developing CAA during acute KD and the persistence of giant CAA during mid-term follow-up, with high sensitivity and specificity. The newly developed KawaCOR score includes age less than 6 months, IVIG initiation after 8 days of fever onset, refractory KD, leucocytosis (>17 × 10^9^), neutrophilia (>16 × 10^9^), thrombocytosis (>400 × 10^9^), anaemia (<103 g/L), hypoproteinaemia (<54 g/L), and hypoalbuminaemia (<32 g/L) as risk factors for CAA in the acute phase, as well as for acute giant and chronic CAA. Unlike in the findings of Son and Huang, CA ectasia on the first echocardiogram did not influence CAA development [1,3,6,7].

Leucocytosis and neutrophilia were the most significant factors influencing our score for the development of acute and chronic CAA. Similar results were presented by Beken [10] and Son et al. [7]. On the other hand, in the Asian population, leucocytosis was not identified as a risk factor [1,3,8], except in the Harada score [11]. Neutrophils play a central role in the pathogenesis of KD. Tsujimoto et al. reported that neutrophil apoptosis is inhibited during the acute phase of KD [12]. Impaired neutrophil apoptosis is observed in infants and younger children, resulting in delayed neutrophil clearance and prolonged inflammation and tissue injury following infections [13], which may explain the increased incidence of CAA in infants, particularly those under 6 months old.

Variations in platelet homeostasis have long been hypothesised to be associated with CAL in patients with KD [14]. According to the regression analysis, a platelet count >400 × 10^9^ increases the risk of CAA development by 4.6 times. Platelet counts <35 × 10^4^/mm^3^ and <300 × 10^9^/L are among the risk factors for IVIG unresponsiveness, as reported by Harada, Egami, and Kobayashi [1].

Haemoglobin < 10.3 g/dL was a risk factor for CAA development in the acute phase. In the North American population, the cutoff value was the same but was not statistically significant in a multivariate regression analysis [7]. Anaemia may be a consequence of increased hepcidin levels. Notably, changes in hepcidin levels after IVIG administration were associated with IVIG treatment resistance and CAL formation, supporting the theory that elevated inflammatory markers and IVIG non-responsiveness may be related to CAL development in KD patients [15].

Hypoproteinaemia and hypoalbuminaemia were among the independent risk factors for CAA development in the acute phase, particularly in cases of giant and chronic CAA. Hypoalbuminaemia is common in KD and may primarily result from increased permeability and albumin leakage during the acute phase [16]—the more pronounced the inflammation, the lower the albumin concentration. Such a severe inflammatory response may also result in elevated CRP levels and a higher WBC count. Interestingly, unlike other studies, we did not find an association between CRP and CAA.

In our cohort, 16.5% of patients developed CAA in the acute phase, and 13 patients developed giant CAA. Calibre pseudonormalisation over time was observed in four patients, while five patients had persistent giant CAA. Two patients underwent surgery, and one patient experienced progression of CAA during follow-up. Progressive giant CAA following KD has rarely been described in the literature [17]. One of these patients developed the largest giant CAA ever described in KD [18]. We found that a WBC count greater than 30 × 10^9^/L and an ANC greater than 23 × 10^9^/L were significantly associated with an increased risk of chronic giant CAA. Additionally, patients with atypical and refractory KD developed chronic CAA more frequently.

To our knowledge, this is the first scoring system that predicts the risk of developing CAA during the acute phase of KD, as well as the risk of giant CAA in both the acute and chronic phases. Furthermore, this is the first scoring system in our region specifically designed to predict the development of CAA. In the KawaCOR score, the cutoff points for predicting acute CAA, giant acute CAA, and chronic giant CAA were set at ≥5, ≥7, and ≥8, respectively, demonstrating notable sensitivity and specificity. The implementation of the KawaCOR score in routine practice could lead to different treatment strategies aimed at preventing CAA development.

The study’s limitations include the retrospective design. Although our research has the largest participant pool among European studies, the number of patients is insufficient to draw definitive conclusions.

## 5. Conclusions

Key factors predicting CAA development included delayed IVIG treatment and certain laboratory markers, such as lower haemoglobin, protein, and albumin levels, as well as higher white blood cell and platelet counts. Notably, a young age (≤6 months) and IVIG initiation after 8 days were independent risk factors. The study also highlighted specific risks for giant and chronic giant CAA, linking them to refractory KD, ongoing inflammation, and low protein and albumin levels.

The KawaCOR score is our region’s first dedicated system to predict CAA development. This scoring system demonstrated excellent sensitivity and specificity in predicting acute CAA and giant CAA, both in the acute phase and during follow-up. The score’s predictive power suggests its potential as a valuable tool for the early identification of high-risk patients, enabling more timely and targeted interventions to mitigate the development and severity of coronary artery complications in Kawasaki disease.

## Figures and Tables

**Table 1:** Differences identified in laboratory analysis regarding the existence of CAA.

	Acute CAA			Giant Acute CAA			Chronic CAA		
	YES	NO	*p*-Value	YES	NO	*p*-Value	YES	NO	*p*-Value
WBC ×10^9^/L	17.7, 13.4–26.5	15.7, 11.5–19	0.01	21, 14.2–31.3	15.8, 11.1–19.4	0.01	30, 20–35.2	15.8, 11.8–19.5	0.01
ANC ×10^9^/L	12.6, 9.1–19.4	10, 6.9–14.2	0.04	16.1, 9.9–23.4	10.5, 6.8–14.5	0.01	22.6, 16–25.9	10.2, 7–14.5	0.005
Plt ×10^9^/L	401, 316–575	334, 252–450	0.009			NS			NS
Hgb g/L	103, 92–108	112, 104–117	<0.001	103, 99–108	111, 103–117	0.04			NS
Protein g/L	54, 52.5–64	63, 57–67	0.05	54, 52–64.0	63, 57–67	0.03			NS
Albumin g/L	34, 32–36	36, 32–39	0.04	31.5, 29–34	36, 33–39	0.005	23.9, 18.6–27	36, 33–39	<0.001

All values are shown as medians with interquartile ranges. Abbreviations: CAA—coronary artery aneurysm, WBC—white blood cell, ANC—absolute neutrophil count, Hgb—haemoglobin, Plt—platelets.

**Table 2:** Univariate and multivariate regression analyses to predict CAA development in the KD acute phase.

	OR	95% CI	*p*-Value
**Univariate Regression Analysis**			
<6 months	9.4	2.9–30.4	<0.001
IVIG initiation > 8 days	6.1	2.2–14.5	0.04
WBC > 17 × 10^9^	3.1	1.3–7.6	0.03
Hgb < 103 g/L	5.6	2.4–15.3	<0.001
Plt > 400 × 10^9^	3.9	1.5–9.9	0.003
Protein < 54 g/L	3.99	1.5–10.7	0.005
**Multivariate Regression Analysis**			
<6 months	17.6	2.5–123.7	0.004
IVIG initiation > 8 days	13.3	2.2–79.4	0.004
Protein < 54 g/L	17.4	2.8–108.9	0.002

Abbreviations: CAA—coronary artery aneurysm, KD—Kawasaki disease, WBC—white blood cell, Hgb—haemoglobin, Plt—platelets.

**Table 3:** Univariate and multivariate regression analyses for predicting giant CAA development in the KD acute phase.

	OR	95% CI	*p*-Value
**Univariate Regression Analysis**			
Refractory KD	4.6	1.4–15.7	0.002
WBC > 21 × 10^9^	5.4	1.7–17.6	0.005
ANC > 16 × 10^9^	4.1	1.2–13.2	0.02
Hgb < 103 (g/L)	4.4	1.4–14.2	0.01
Protein < 54 × 10^9^	7.3	2.1–25.2	0.002
Albumin < 32 g/L	4.6	1.7–15.7	0.01
**Multivariate Regression Analysis**			
Refractory KD	5.1	1.16–22.5	0.03
Hgb < 103 (g/L)	3.9	0.9–17.2	0.04

Abbreviations: CAA—coronary artery aneurysm, KD—Kawasaki disease, WBC—white blood cell, ANC—absolute neutrophil count, Hgb—haemoglobin.

**Table 4:** Univariate regression analysis for predicting giant CAA development in the KD acute phase.

	OR	95% CI	*p*-Value
Univariate Regression Analysis			
Refractory KD	11.2	1.2–103.5	0.03
WBC > 30 × 10^9^	34	4.7–243.1	<0.001
ANC > 23 × 10^9^	34.7	4.8–248	<0.001
Albumin < 23 g/L	6.3	0.99–39.7	0.05

Abbreviations: CAA—coronary artery aneurysm, KD—Kawasaki disease, WBC—white blood cell, ANC—absolute neutrophil count.

**Table 6:** Univariate regression analysis for predicting acute CAA, giant acute, and chronic CAA based on the KawaCOR score.

	OR	95% CI	*p*-Value
Univariate Regression Analysis			
Acute CAA score ≥ 5	8.7	3.4–22.6	< 0.001
Acute giant CAA score ≥ 7	25.7	6.7–100.3	< 0.001
Giant chronic CAA score ≥ 8	71.5	8.5–597.7	< 0.001

Abbreviation: CAA—coronary artery aneurysm.

## Data Availability

The original contributions presented in this study are included in the article/Supplementary Material. Further inquiries can be directed to the corresponding author.

## Supplementary Materials

The following supporting information can be downloaded at https://www.mdpi.com/article/10.3390/life16040607/s1. Figure S1: Kawasaki disease scoring systems by country of development. Figure S2: Dynamics of coronary artery aneurysm changes after Kawasaki disease during the follow-up period. Abbreviations: CAA—coronary artery aneurysm, pt—patient, pts—patients. Figure S3: Results of power analysis in G*Power 3.1.

## Abbreviations

AHA	American Heart Association
AUC	Area under the curve
CA	Coronary artery
CAL	Coronary artery lesions
CAA	Coronary artery aneurysm
IVIG	Intravenous immunoglobulin
IQR	Interquartile range
KD	Kawasaki disease
LM	Left main
LAD	Left anterior descending
LCx	Left circumflex
RCA	Right coronary artery
WBC	White blood cell
ANC	Absolute neutrophil count
Hgb	Haemoglobin
NLR	Neutrophil-to-lymphocyte ratio
Plt	Platelets
OR	Odds ratio
CI	Confidence interval
