# Supplementary material for: Comprehensive Assessment of the Risk of Developing Coronary Artery Aneurysm in Kawasaki Disease: A KawaCOR Score Study
Source: Life (Basel). 2026 Apr 7;16(4):607. doi: 10.3390/life16040607 (PMC13117544; doi:10.3390/life16040607)
Supplement: Supplementary file 1 [file life-16-00607-s001.zip › life-4184262-supplementary.pdf]

Supplementary Materials

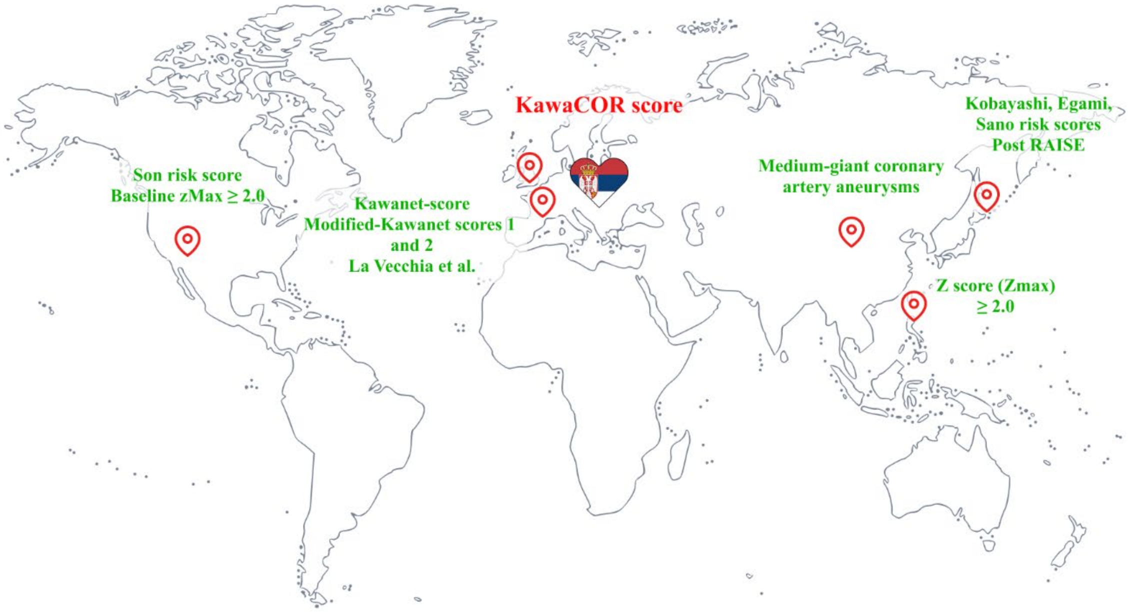

Supplement Figure S1. Kawasaki disease scoring systems vary by country of development.

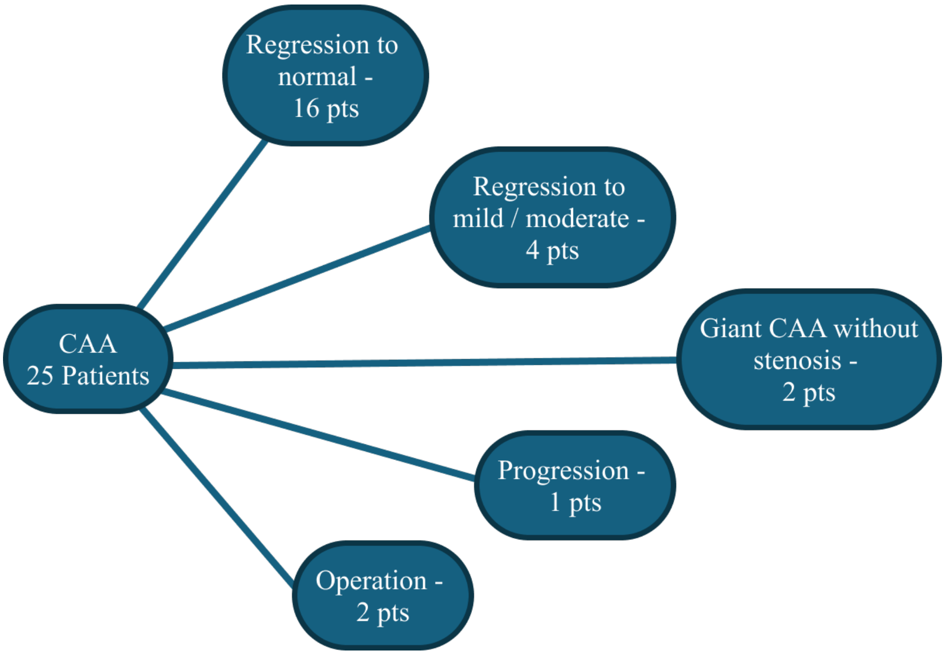

Supplement Figure S2. Dynamics of coronary artery aneurysm changes after Kawasaki disease during the follow-up period

Abbreviations: CAA - coronary artery aneurysm, pt – patient, pts - patients

Results of power analysis in G*Power 3.1

z tests - Proportions: Difference between two independent proportions

**Analysis:**	Post hoc: Compute achieved power		
**Input:**	Tail(s)	=	Two
	Proportion p2	=	0.35
	Proportion p1	=	0.10
	α err prob	=	0.05
	Sample size group 1	=	75
	Sample size group 2	=	76
**Output:**	Critical z	=	0.1333849
	Power (1-β err prob)	=	0.9641345

(Power = 0.964)

Supplement Figure S3. Results of power analysis in G*Power 3.1
